# Comparison of Foam and Liquid Sclerotherapy for the Treatment of Lower Extremity Varicose Veins and Telangiectasia in Obese Patients

**DOI:** 10.7759/cureus.42571

**Published:** 2023-07-27

**Authors:** Eyüp Murat Kanber

**Affiliations:** 1 Department of Cardiovascular Surgery, Istanbul Private Clinic, Istanbul, TUR

**Keywords:** telangiectasia, obese, lower extremity, liquid sclerotherapy, foam sclerotherapy

## Abstract

Objective: Sclerotherapy is an accepted treatment modality for reticular varicose veins and telangiectasia. We aimed to compare the success and safety of foam and liquid sclerotherapy in obese patients with lower extremity varicose veins and telangiectasia.

Methods: The present study was performed in a non-randomized prospective manner, and obese patients with lower extremity varicose veins (patients with anatomy and pathophysiology classification {CEAP} class C1) and telangiectasia treated with foam sclerotherapy and liquid sclerotherapy were enrolled into the study. Patients treated with foam sclerotherapy and liquid sclerotherapy were compared with regard to preoperative parameters, procedure success, Visual Analog Score (VAS), patient satisfaction, and complications.

Results: The VAS scores at the first hour and sixth hour were statistically higher in the foam sclerotherapy group (p=0.001 and p=0.001, respectively). The success rate after the first session was 88.2% in the foam sclerotherapy group and 69.4% in the liquid sclerotherapy group (p=0.008). After all sessions, the success rates were similar between groups (p=0.607). The foam sclerotherapy group required an average of 1.1 sessions, while the liquid sclerotherapy group required 1.4 sessions (p=0.001). At the third-month follow-up, the success rate was significantly higher in the foam sclerotherapy group than liquid sclerotherapy group (91.2% and 77.4%; p= 0.030). In the foam sclerotherapy group, 80.9% of the patients were very satisfied, while this rate was 58.1% in the liquid group (p=0.012). The rates of ecchymosis and hyperpigmentation in the first week after the procedure were significantly higher in the foam sclerotherapy group (p=0.003 and p=0.040, respectively).

Conclusion: Our findings showed that foam sclerotherapy had a significantly higher success rate after the first session, and third month follow-up with higher patient satisfaction. In contrast, liquid sclerotherapy had significantly lower VAS scores in the first and sixth hours following the procedure and had lower ecchymosis and hyperpigmentation in the first week after the procedure.

## Introduction

Lower extremity chronic venous insufficiency (LECVI) is associated with inadequate blood flow from the lower extremity veins to the heart, pathological dilatation of lower extremity veins, and abnormal blood stasis in veins [1]. Previous reports demonstrated that the prevalence of LECVI is increasing, and almost one-third of women and one-fifth of men face LECVI and LECVI-related complications [2]. Lower extremity cramps, fatigue, swelling, weight gain, restless leg, and even deep vein thrombosis and pulmonary embolism could be seen in patients with untreated LECVI. Telangiectasias are commonly detected in patients with LECVI and lead to cosmetic complaints beyond the aforementioned complications [3]. Treatment of LECVI is more complicated in specific populations, including elderly patients, patients with severe health insufficiency, and obese patients [4].

Sclerotherapy is an accepted treatment modality for reticular varicose veins and telangiectasia, and endothelial damage to veins and the inflammatory process in sclerotherapy play roles in the healing of varicose veins and telangiectasia [5]. Previous studies investigated the efficiency and safety of different medical agents for the management of reticular varicose veins and telangiectasia of the lower extremity, and the efficiency of liquid sclerotherapy and foam sclerotherapy was proven. Kaygin and Halici applied foam and liquid sclerotherapy in patients with lower extremity varicose veins, and the authors stated that both foam and liquid sclerotherapy were safe treatment options with acceptable complications [6]. In another study, Hamahata et al. achieved higher success with foam sclerotherapy in patients with lower extremity varicose veins [7]. However, Park and colleagues faced a higher complication rate with liquid sclerotherapy in the same study population [8].

Although previous papers focused on the efficiency and safety of foam and liquid sclerotherapy in patients with LECVI, no study compared the role of foam and liquid sclerotherapy in obese patients with lower extremity varicose veins and telangiectasia. In the present study, the aim was to compare the success and safety of foam and liquid sclerotherapy in obese patients with lower extremity varicose veins and telangiectasia.

## Materials and methods

The present study was performed in a non-randomized prospective manner between January 2021 and December 2002, and obese patients with lower extremity varicose veins and telangiectasia treated with foam sclerotherapy and liquid sclerotherapy were enrolled in the study. All patients were informed in detail about their illness, treatment options, treatment success, possible complications, and follow-up periods. Informed consent was obtained 24 hours before the procedure, and the study was done in accordance with the Helsinki Declaration of human rights. The definition of obesity was accepted as World Health Organization criteria, and patients with body mass index (BMI) >30 kg/m^2^, were accepted as obese. Non-obese patients, patients who received additional treatments for lower extremity venous insufficiency, patients aged ≤18 years, immobile patients, patients with clinical, etiology, patients with anatomy and pathophysiology classification (CEAP) other than class C1, and patients with local infection around the application area, and/or systemic infection were excluded from the study. Also, patients with poor health conditions, with malignancy in the same extremity, and who underwent radiotherapy for the same extremity were not enrolled in the study.

The medical history of patients was noted, and all patients underwent detailed physical examinations. For each patient, color Doppler ultrasonography was done for the lower extremity. Also, the type of procedure, the procedure's success after the first session, requirements for additional sessions, success after all sessions, and success of the procedure in the third-month follow-up were noted. Patient age, sex, BMI, side of procedure, diameter of vein, and Venous Clinical Severity Score (VCSS) were recorded. Patient Visual Analog Scores (VAS) were noted in the first hour, sixth hour, and first^ ^week, and patient satisfaction levels (very satisfied, satisfied, neutral, and dissatisfied) were recorded.

Foam sclerotherapy and liquid sclerotherapy

All procedures were done using 30 G needles. Foam sclerotherapy was performed according to the Tessari method, and 0.5%-1% polidocanol solution at the maximum dose of 0.5 ml was administered to each vein [9]. A maximum 2 mg/kg total dose was administered while the leg was elevated at 45°-60°, and after the administration, the leg was rested for 15 minutes. Also, liquid sclerotherapy was performed using 1% polidocanol while patients were lying down. For all patients, compression was provided by elastic bandages, and compression therapy continued for a week.

To clarify the efficiency and safety of foam sclerotherapy and liquid sclerotherapy in obese patients with lower extremity reticular varicose veins and telangiectasia, patients treated with foam sclerotherapy and liquid sclerotherapy were compared with regard to preoperative parameters, procedure success, VAS score, patient satisfaction, and complications.

Statistical analysis

The Statistical Package for the Social Sciences (SPSS), version 27.0 (IBM Corp., Armonk, NY) was used for statistical analysis. The normality of variable distribution was evaluated with the Shapiro-Wilk test. Student's t-test was used to compare normally distributed data. Data that did not show normal distribution were compared with the Mann-Whitney U test. A Chi-square test was performed to compare rates between the two groups. Finally, p values are shown as two-sided statistical tests with a statistical significance of p <0.05.

## Results

Demographic data for the patients were compared between the groups, as shown in Table 1. The mean age of the patients was 42.2 years in the foam sclerotherapy group and 40.1 years in the liquid sclerotherapy group (p=0.242). Gender, BMI, procedure side, and VCSS were statistically similar between the groups (p= 0.455, p= 0.109, p= 0.841, and 0.406, respectively). The mean vein diameter was 2.0 in the foam sclerotherapy group and 1.9 in the liquid sclerotherapy group (p=0.341).

**Table 1:** Comparison of demographic data between groups * mean ± standard deviation, VCSS: Venous Clinical Severity Score

	Foam sclerotherapy (n:68)	Liquid sclerotherapy (n:62)	p-value
Age (years)*	42.2±9.7	40.1±10.9	0.242
Sex, n (%)			0.455
Male	47 (69.1%)	39 (62.9%)	
Female	21 (30.9%)	23 (37.1%)	
Body mass index (kg/m^2^)	34.6±2.6	35.3±2.8	0.109
Side of procedure, n (%)			0.841
Right	35 (51.5%)	33 (53.2%)	
Left	33 (48.5%)	29 (46.8%)	
Diameter of the vein (mm)*	2.0±0.8	1.9±0.8	0.341
VCSS *	6.1±1.6	5.4±1.8	0.406

The VAS scores at the first and sixth^ ^hours were statistically higher in the foam sclerotherapy group (p=0.001 and p=0.001, respectively). However, there was no statistical difference in the first^ ^week's VAS scores between the groups (p=0.465). The success rate after the first session was 88.2% in the foam sclerotherapy group and 69.4% in the liquid sclerotherapy group (p=0.008). After all sessions, the success rates were similar between groups (p=0.607). The foam sclerotherapy group required an average of 1.1 sessions, while the liquid sclerotherapy group required 1.4 sessions (p=0.001). At the third-month follow-up, the success rate was significantly higher in the foam sclerotherapy group (91.2% and 77.4%; p= 0.030). In the foam sclerotherapy group, 80.9% of the patients were very satisfied, while this rate was 58.1% in the liquid group (p=0.012) (Table 2).

**Table 2:** Comparison of post-procedural data between groups * mean ± standard deviation, VAS: Visual Analog Score

	Foam sclerotherapy (n:68)	Liquid sclerotherapy (n:62)	p-value
VAS score*			
1^st ^hour	3.8±1.2	2.3±1.0	0.001
6^th ^hour	2.5±1.0	1.5±0.6	0.001
1^st^ week	0.3±0.2	0.2±0.1	0.465
Success, n (%)			
After 1^st ^session	60 (88.2%)	43 (69.4%)	0.008
Number of sessions required	1.1±0.3	1.4±0.6	0.001
After all sessions	65 (95.6%)	58 (93.5%)	0.607
3 months after last session	62 (91.2%)	48 (77.4%)	0.030
Patient satisfaction, n (%)			0.012
Very satisfied	55 (80.9%)	36 (58.1%)	
Satisfied	9 (13.2%)	10 (16.1%)	
Neutral	3 (4.4%)	12 (19.4%)	
Dissatisfied	1 (1.5%)	4 (6.5%)	

The complication rates in the first week and third month after the procedure are compared in Table 3. The rates of ecchymosis and hyperpigmentation in the first week after the procedure were significantly higher in the foam sclerotherapy group (p=0.003 and p=0.040, respectively). However, there were no significant differences in ecchymosis and hyperpigmentation rates between the groups third months after the procedure (p=0.564 and p=0.681). In the foam sclerotherapy group, paresthesia was detected in 2.9% of patients in the first week and 1.5% in the third-month post-procedure. In the liquid sclerotherapy group, the paresthesia rate was 1.6% in both periods. Skin necrosis developed in 3.2% of patients in the liquid sclerotherapy group, while no skin necrosis was detected in the foam group.

**Table 3:** Comparison of post-procedural complications between groups NA: Not Available

	Foam sclerotherapy (n:68)	Liquid sclerotherapy (n:62)	p-value
Ecchymosis, n (%)			
1^st^ week	44 (64.7%)	24 (38.7%)	0.003
3^rd^ month	10 (14.7%)	7 (11.3%)	0.564
Paresthesia, n (%)			NA
1^st^ week	2 (2.9%)	1 (1.6%)	
3^rd^ month	1 (1.5%)	1 (1.6%)	
Hyperpigmentation, n (%)			
1^st^ week	34 (50.0%)	20 (32.2%)	0.040
3^rd^ month	14 (20.6%)	11 (17.7%)	0.681
Thrombophlebitis, n (%)			NA
1^st^ week	3 (4.4%)	1 (1.6%)	
3^rd^ month	1 (1.5%)	-	
Skin necrosis, n (%)	-	2 (3.2%)	NA

## Discussion

Obesity has become a pandemic in the last century, and almost one-third of the world’s population is affected by obesity and obesity-related complications [10]. Previous reports also demonstrated that lower extremity venous insufficiency is more common in the obese population, and treatment of lower extremity venous insufficiency is challenging and complicated in obese patients [11]. Moreover, some authors claimed that the success of cardiovascular procedures decreases with obesity. Thus, for the first time, we conducted a prospective study comparing foam and liquid sclerotherapy in obese patients with reticular varicose veins and telangiectasia in the lower extremities. Foam sclerotherapy had a significantly higher success rate after the first session and at third-month follow-up, and higher patient satisfaction. In contrast, liquid sclerotherapy had significantly lower VAS scores in the first and sixth hours following the procedure and had lower ecchymosis and hyperpigmentation one week after the procedure.

Successful treatment of reticular varicose veins and telangiectasia of the lower extremity is the main goal of all types of sclerotherapy regimens. Park and colleagues investigated foam sclerotherapy and liquid sclerotherapy in lower extremity varicose veins, and the authors achieved 92.7% success with foam sclerotherapy and 71.8% success with liquid sclerotherapy, which was significantly better in favor of foam sclerotherapy [8]. In another study by Yamaki et al., who analyzed foam sclerotherapy for 37 patients and liquid sclerotherapy for 40 patients, foam sclerotherapy had a significantly higher success rate for managing lower extremity varicose veins [12]. In accordance with the aforementioned studies, a significantly higher success rate was achieved with foam sclerotherapy in obese patients with lower extremity varicose veins.

Pain and patient discomfort following sclerotherapy can deteriorate patient quality of life. Darvall and colleagues analyzed the expectations of 351 patients who underwent foam sclerotherapy for lower extremity venous insufficiency, and they concluded that up to 25% of patient expectations were not met [13]. Also, Kaygin and Halici stated that foam sclerotherapy was a more painful procedure compared to liquid sclerotherapy; however, the difference was not statically significant [6]. In contrast, Moser et al. used foam sclerotherapy and liquid sclerotherapy for hemorrhoidal disease and found that patient satisfaction was significantly higher with foam sclerotherapy [14]. In the present study, we achieved significantly higher patient satisfaction with foam sclerotherapy in obese patients with lower extremity venous disease, in spite of higher VAS scores at the first hour and sixth hour.

Previous studies reported most complications following sclerotherapy management are minor and temporary. Also, previous reports that compared foam sclerotherapy and liquid sclerotherapy with regard to complications found controversial results. Jia et al. reviewed articles about foam sclerotherapy and the authors found 17.8% had skin pigmentation and 25.6% had pain at the injection site [15]. Another meta-analysis by Bi and colleagues found no significant difference between foam sclerotherapy and liquid sclerotherapy for pain, local inflammation, and hyperpigmentation [16]. We found that ecchymosis and hyperpigmentation rates were significantly higher in obese patients treated with foam sclerotherapy.

The relatively small patient number in the study could be accepted as a limitation. Only short-term results of foam and liquid sclerotherapy were noted, and the study lacks long-term results in obese patients. In addition, the study is a single-center study. Finally, the cost of procedures was not analyzed in the present study, which may be a subject for further studies.

## Conclusions

Our study for the first time revealed that both foam sclerotherapy and liquid sclerotherapy are effective and safe treatment options for lower extremity varicose veins in obese patients. In addition, our findings showed that foam sclerotherapy had a significantly higher success rate after the first session and third-month follow-up with higher patient satisfaction. In contrast, liquid sclerotherapy had significantly lower VAS scores in the first and sixth hours following the procedure and had lower ecchymosis and hyperpigmentation in the first week after the procedure.
